# Intestinal Absorption of Lipids Using a Pancreatic Enzyme-Free Nutritional Supplement in Patients with Cystic Fibrosis: A Randomized, Double-Blind, Crossover Pilot Trial

**DOI:** 10.3390/nu14030680

**Published:** 2022-02-05

**Authors:** Tara L. Barto, Clarissa F. Morency, Nicoline Schaap, Ami B. Patel, Daniel J. Monticello

**Affiliations:** 1 Department of Medicine, Baylor College of Medicine, Houston, TX 77030, USA; clarissa.morency@bcm.edu (C.F.M.); s_nicoline@hotmail.com (N.S.); ami.patel@bcm.edu (A.B.P.); 2 GlycosBio Food Sciences, Houston, TX 77021, USA; dmonticello@glycosbio.com

**Keywords:** nutritional supplement, cystic fibrosis, PERT-free, pancreatic enzyme, enzyme modified oil, exocrine pancreatic insufficiency

## Abstract

Nutritional supplements for patients with exocrine pancreatic insufficiency (EPI) typically utilize pancreatic enzyme replacement therapy (PERT) which is associated with gastrointestinal side effects. We evaluated serum triglyceride levels in patients with cystic fibrosis following consumption of an enzyme-modified oil oral nutritional supplement (EMO-ONS) versus a standard triacylglycerol-based ONS product (TAG-ONS) used concomitantly with PERT and patient tolerability between the two approaches. Ten subjects with CF and EPI taking PERT were enrolled in a single-center, double-blind, cross-over proof of concept trial. Five subjects randomized to Arm 1 were administered a PERT placebo and EMO-ONS and 5 subjects in Arm 2 were administered TAG-ONS+PERT. After 4 to 14 days, subjects received the opposite ONS. Serum triglyceride levels were measured at baseline and hourly for 6 h. Following the above, subjects were randomly assigned to receive 2 daily servings of EMO-ONS+PERT placebo or TAG-ONS+PERT at home for 7-days, self-reporting gastrointestinal symptoms daily. Mean change in peak serum triglyceride levels were similar for both groups (EMO-ONS = 41.9 ± 46.7 mg/dL vs. TAG-ONS+PERT = 46.4 ± 44.1 mg/L; *p* = 0.85). There was no difference in mean ratio of the serum triglyceride AUC between the two groups (*p* = 0.58) or self-reported gastrointestinal tolerance. EMO-based products may provide a PERT-free alternative to traditional ONS products in patients with cystic fibrosis.

## 1. Introduction

Cystic fibrosis (CF) is a debilitating autosomal recessive genetic disorder that affects over 30,000 individuals in the United States [1]. CF is a result of mutations in the gene for the CF transmembrane conductance regulator (CFTR) protein [2]. It is manifest primarily as an accumulation of mucus in the lungs and pancreas. In the lungs, this results in a vicious cycle of infection and of inflammation that ultimately leads to respiratory failure. In the pancreas, this thick mucus blocks the release of digestive enzymes required for the breakdown of fats and oils (lipids), proteins, and carbohydrates.

The life expectancy and quality of life of individuals with CF has increased dramatically in the last three decades. Mortality rates in the U.S. have decreased from 2.3 deaths per 100 individuals in 1988 to 1.2 deaths per 100 individuals in 2019, with the median predicted survival age for individuals born in 2019 being 48.4 years [1]. This improvement is a result of the development of CFTR modulators, advancement in the treatment of lung disease and reductions in recurrent infections, and better management of nutrition [3,4,5]. Maintaining adequate nutritional status has been shown to be directly associated with improved pulmonary function and survival in both pediatric and adult patients with CF [5].

Chronic deficiency in the secretion of digestive enzymes by the pancreas is termed exocrine pancreatic insufficiency (EPI). EPI can result in malnutrition due to a reduced amount of pancreatic enzyme being delivered to the small intestine with potential associated increases in both morbidity and mortality [6]. The degree of EPI varies significantly from patient to patient, and even in a single patient from day to day [7]. EPI is one the most common manifestations of CF with between 80% and 90% of individuals developing this condition [8]. EPI is often under-diagnosed and under-reported because of the wide spectrum and extent of symptoms and the lack of reliable diagnostic tests [9].

The effects of EPI can be mitigated by consuming pancreatic enzyme capsules, otherwise known as pancreatic enzyme replacement therapy (PERT). PERT consists of capsules that contain a mixture of lipases, proteases, and carbohydrases produced by extracting digestive enzymes from pig pancreases. Patients using PERT must take capsules before, during, and after meals since these enzymes are needed throughout the digestive process. The failure to consume these capsules often leads to abdominal discomfort, bloating, and steatorrhea [7,10]. Since PERT often requires patients to take 10 to 15 enzyme capsules per day, compliance can be problematic, with an overall adherence rate for taking pancreatic enzymes reported at less than 50% [11,12]. As a result, identifying a PERT-free alternative to nutritional supplementation for patients with cystic fibrosis can potentially lead to improved nutritional status and a reduction in the risk of hospitalizations associated with non-adherence to PERT [13].

The aim of the current study was to evaluate a new PERT-Free nutritional supplement for patients with CF and an EPI diagnosis to determine if it could be used as an alternative to the use of PERT in combination with a standard nutritional supplement.

## 2. Materials and Methods

**Figure 1:**
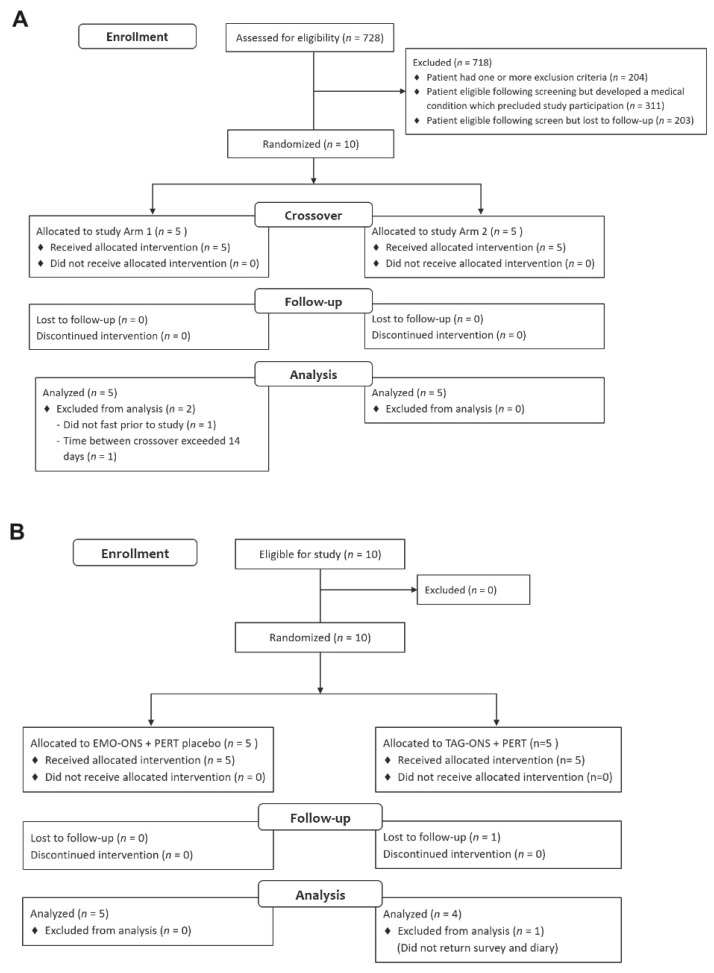
CONSORT (CONsolidated Standards of Reporting Trials) flow diagram for (**A**) Phase 1; and (**B**) Phase 2 of the trial. EMO-ONS: enzyme-modified oil oral nutritional supplement. PERT: pancreatic enzyme replacement therapy. TAG-ONS: triacylglycerol-based oral nutritional supplement.

Ten subjects with confirmed CF and documented EPI utilizing PERT were enrolled and participated in the present proof of concept study between 31 August 2018 and 31 May 2019. The study is registered at Clinicaltrials.gov (registration number NCT04966897). Potential study subjects were identified based on screening of patient records for those being treated for CF at either the Maconda Brown O’Connor, Ph.D., Adult Cystic Fibrosis Center at Baylor College of Medicine or the Cystic Fibrosis Care Center at Texas Children’s Hospital. The inpatient portion of the study was conducted at the Clinical Research Center (CRC) at Texas Children’s Hospital. Patients who met the study eligibility criteria and not excluded for other reasons which precluded study enrollment were approached for participation. Figure 1A,B outlines the Consolidated Standards of Reporting Trials (CONSORT) diagram for the two phases of the study. The protocol was approved by the Western IRB (WIRB) and the Baylor College of Medicine IRB and informed written consent was obtained from all study participants prior to enrollment or from a parent for subjects who were minors.

### 2.1. Trial Design

**Figure 2:**
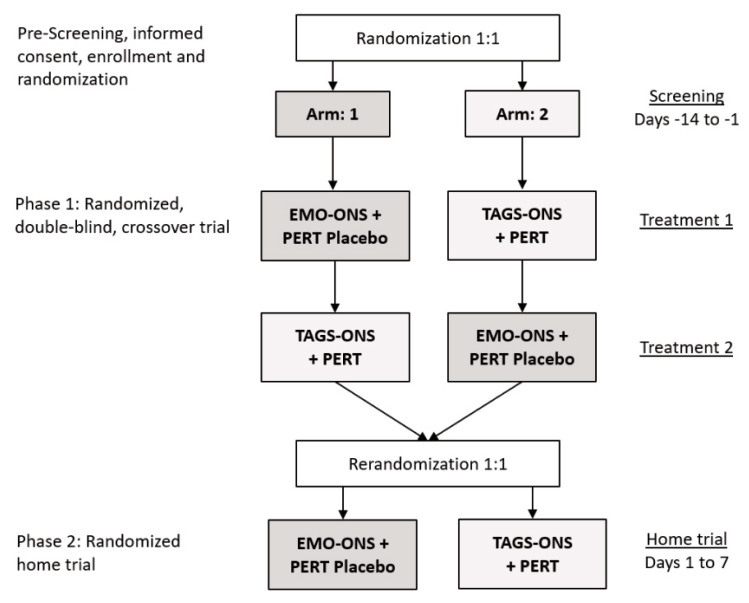
Study Design. EMO-ONS: enzyme-modified oil oral nutritional supplement. PERT: pancreatic enzyme replacement therapy. TAG-ONS: triacylglycerol-based oral nutritional supplement.

This was a double-blind, randomized, cross-over, placebo-controlled, interventional pilot study of 10 patients with CF (Figure 2). The study was divided into inpatient (Phase 1) and outpatient (Phase 2) phases with randomization of the study participants prior to Phase 1 and re-randomization prior to the start of Phase 2. Inclusion criteria were patients (1) previously diagnosed with CF aged 12 years or older who were currently receiving treatment with a commercially available pancreatic enzyme product for more than 3 months, (2) in clinically stable condition without evidence of acute respiratory disease within 1 month of enrollment, (3) had a stable body weight defined as no more than 5% decline within 3 months of enrollment, (4) if female and of child-bearing potential, agree to continue using a medically acceptable method of birth control throughout the study and for 30 days after participation in the study, (5) have the ability to take oral medication and oral liquid nutritional supplements and be willing to adhere to the study interventions, and (6) agree to adhere to lifestyle considerations throughout the duration of the study, including abstaining from alcohol and vigorous exercise for 24 h before Treatments 1 and 2 until after collection of the final blood sample.

Exclusion criteria included the presence of respiratory, gastrointestinal/hepatic, or metabolic/nutritional disease except if these were underlying conditions of CF or EPI. Individuals with diseases of the cardiovascular, urogenital, hematologic/immunologic, head, ears, eyes, nose, throat, dermatologic/connective tissue, musculoskeletal, and endocrine (except controlled diabetes mellitus) areas, and neurologic/psychiatric illness; milk, nut or soy allergies; recent major surgery; or other relevant diseases as revealed by history, physical examination, and/or laboratory assessments, which could limit participation in or completion of the study were excluded. Additional exclusion criteria included a history of abdominal or intestinal disease or procedures, receiving enteral tube feeds for ≥50% of daily calorie intake, pregnancy or lactation, known allergy to pancreatin or inactive ingredients (excipients) of pancreatin capsules, suspected non-compliance or non-cooperation, intake of experimental drugs within 30 days prior to study start, mental disability, or any other lack of fitness in the investigator’s opinion that would preclude subject’s participation in or ability to complete the study. Previous diagnosis of human immunodeficiency virus; being listed for lung transplantation, other solid organ transplant or documented forced expiratory volume (FEV) ≤25%; use of lipid lowering therapy including statins, fibrates, niacin, and proprotein convertase subtilisin kexin type 9 (PCSK9) inhibitors that cannot be held at least 14 days prior to Day 1 and through day 15 of the study were further exclusion criteria.

### 2.2. Nutritional Supplements

**Figure 3:**
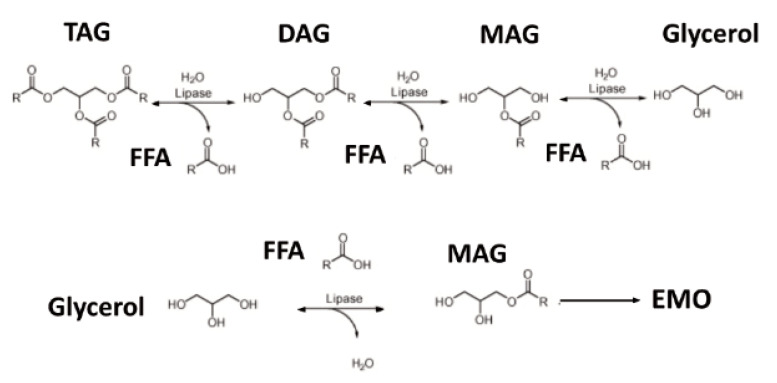
Process for producing enzyme modified oil. DAG: diacylglyceride. EMO: enzyme modified oil. FFA: free fatty acid. MAG: monoacylglyceride. TAG: triacylglycerides.

The investigational oral nutritional supplement (ONS) utilized for the current study was a proprietary enzyme modified oil (EMO) oral nutritional supplement (EMO-ONS) developed by the study sponsor (GlycosBio Inc., Houston, TX, USA). EMO-ONS is a ready-to-drink nutritional supplement developed for individuals with EPI intended for consumption without PERT. EMO-ONS ready-to-drink shake formulation includes a source of lipids, protein equivalents (amino acids), carbohydrates (simple sugars), vitamins, minerals, and fiber in addition to the traditional surfactants, flavoring, and stabilizing agents typically found in over-the-counter food products. The lipid (fat) in EMO-ONS was provided predominantly in the form of monoacylglycerols (MAG) produced by the study sponsor using a GRAS (generally recognized as safe) enzymatic process (Figure 3). MAGs and fatty acids are the normal product of pancreatic lipase activity in the small intestine and they are readily absorbed by enterocytes without the need of further digestion. The “Enzyme Modified Oil” was produced enzymatically from almond oil and incorporated at 7.4% in the product. The control nutritional supplement “TAG-ONS” was a commercially available product (Boost Plus^®^, Nestlé Health Science, Bridgewater, NJ, USA) with lipid content (as triacylglycerols) of 5.9%.

### 2.3. Experimental Protocol

Phase 1. Participants were randomly assigned to one of two study arms (Figure 2). Block randomization was used to assign patients to each of the study arms. Separate randomization lists were prepared in order to stratify the groups by sex. Subjects were randomly assigned to either the EMO-ONS or TAG-ONS arm first (Treatment 1) and then crossed over to the other arm for the crossover phase of the study (Treatment 2). The randomization lists were provided to the unblinded pharmacist who prepared the appropriate blinded product (ONS and capsules) for each treatment period of the study. Identical bottles were used for the two ONS products and the PERT and PERT placebo capsules were also similar.

After an overnight fast, all subjects came to the clinic and underwent a physical exam that included vital signs, weight, and concomitant medications record and blood testing for a baseline blood chemistry analysis which included a comprehensive metabolic panel (CMP) and a standard lipid panel. An assessment of stool frequency and consistency, bloating, and abdominal pain was also performed.

Following the above, patients randomized to Arm 1 were administered the EMO-ONS over a 20-min time period in a volume sufficient to supply 0.5 g of lipid per kg of body weight and a PERT placebo. Patients in Arm 2 were administered the standard nutritional supplement (TAG-ONS) in a volume sufficient to supply 0.5 g of lipid per kg of body weight over the same time period plus PERT capsules (24,000 iu/capsule) at a minimum dose to ensure 2500 iu of lipase activity per gram of fat ingested, depending on body weight. Identical PERT and placebo capsule were prepared by repackaging CREON^®^ (pancrelipase) or an inert filler into new white capsules (Doyles Pharmacy, Houston, TX, USA). Subjects with a body weight of 56 kg or less received 3 capsules and subjects with a body weight above 56 kg received 4 capsules. PERT and PERT placebo capsules were administered just prior to the subject drinking the ONS. The entire drink was consumed in-clinic over 20 mins. All study subjects, investigators, and associated individuals were blinded to which ONS and capsule (placebo or PERT) the subjects received.

Blood samples were drawn for both arms of the study at hourly intervals after ingestion of the above for 6 h in order to perform a standard lipid panel and test for glucose levels for each time interval. A CMP was also performed for blood drawn at 6 h. Patients were permitted to consume water during the 6-h study period. A patient tolerability survey was completed at the end of the study period. Adverse events, if encountered, were recorded accordingly to Common Terminology Criteria for Adverse Events (CTCAE) v4.03.

After a period of no fewer than 4 days and no more than 14 days and an overnight fast, all subjects returned to the clinic for the cross-over phase of the study. Subjects were administered the opposite nutritional therapy with the subjects in Arm 1 administered TAG-ONS plus PERT and subjects in Arm 2 receiving EMO-ONS plus a PERT placebo capsule. All of the study conditions, measurements, blood draws, and dosing parameters were the same as the above.

Phase 2. Subjects enter this phase of the study in a home setting immediately following completion of Phase 1. Study participants were re-randomized using block randomization (Figure 2) as with Phase 1 and assigned to Arm 1 where patients received EMO-ONS (17.5 g EMO) plus a PERT placebo capsule, or Arm 2 where patients received TAG-ONS (14 g of TAG per serving) plus PERT capsules. The ONS were taken as a fixed 237 mL (8 ounces) twice daily as a snack between breakfast and lunch and also between lunch and dinner. The PERT dosage used for Phase 2 of the study was to ensure a minimum of 2500 iu of lipase activity per gram of fat ingested, which equated to 2 capsules per ONS serving. Subjects were asked to take the capsules just prior to consuming the drink. Study product was packaged and labeled as either treatment A or B to maintain the study blind. Study personnel provided the appropriate labeled ONS and capsules to the study participants based on the randomization list. Subjects were asked to return empty bottles and any remaining capsules upon completion of Phase 2 of the study.

Participants were asked to complete a palatability and tolerability Survey on a daily basis on days 1 through 7 of the at-home portion of the study and to record what time they ate their last meal prior to taking the ONS and capsules, the time they consumed the ONS, and how many capsules they took.

### 2.4. Study Endpoints

The primary endpoints for the study were the change from baseline for the maximum serum triglyceride concentration following administration of the ONS during Phase 1 of the study, time to maximum serum triglyceride concentration, and the incremental area under the curve (AUC) for triglyceride serum levels over 6 h following ingestion of the ONS. Change in serum triglyceride levels was utilized as an endpoint since it was anticipated that initial serum levels would vary considerably from patient to patient and between visits.

Secondary endpoints for Phase 1 of the study were changes in serum glucose, total cholesterol, HDL cholesterol, LDL cholesterol, and VLDL cholesterol using the same measures at the above and incidence of gastrointestinal (GI) symptoms, including nausea, heartburn, abdominal pain, steatorrhea, bloating and other reported symptoms. Secondary endpoints for Phase 2 of the study included average daily number of stools and the percentage of days with (1) hard or formed/normal or soft stools, (2) no steatorrhea, (3) no abdominal pain, and (4) no bloating. Safety endpoints were assessed by comparing differences between the study arms following systematic assessment of adverse events according to CTCAE v4.03 and changes in blood chemistry or vital signs. Differences in the palatability of each ONS was an exploratory endpoint and assessed via a participant survey.

### 2.5. Statistical Analysis

Data was summarized using descriptive statistics (continuous data) and/or contingency tables (categorical data) for demographic and baseline/screening characteristics, questionnaires, efficacy measurements, safety measurements, and all relevant measurements. Categorical data was presented as frequencies and percentages. For continuous data, mean and standard deviations were calculated. The incremental or decremental serum lipid concentrations (triglycerides, total cholesterol, LDL, HDL, vLDL) was calculated by subtracting baseline (time 0) from postprandial values (baseline value becomes 0) and compared using Wilcoxon rank sum test. Absorption was assessed by the area under the curve of the incremental/decremental concentration time prolife as calculated by the trapezoidal method for each individual for Treatments 1 and 2. The incremental and total AUC in treatment Arm 1 were compared descriptively to treatment Arm 2 using a matched pairs approach. Differences in AUCs for markers of lipid absorption were descriptively described using parametric or nonparametric matched pairs analysis depending on the distribution of the data.

## 3. Results

### 3.1. Crossover Study

Table 1 lists the baseline patient characteristics and treatment provided for the ten subjects who participated in the study. Mean subject age was 20.6 ± 28.2 years (range 12.4–49.5 years) with 7 males and 3 females. There were no significant differences in the baseline characteristics between the two arms of the clinical portion of the study. Two patients were excluded from analysis. This included one patient who was excluded because of the time between treatment 1 and treatment 2 exceeding 14 days due to illness. A second patient was excluded as a result of the likelihood that the patient did not fast (had breakfast) prior to both treatments. This hypothesis was supported by a lack of a change in triglyceride levels following ingestion of either EMO-ONS or TAG-ON and an increase in serum glucose at the end of each test period. Both excluded patients were in Arm 1 of the study. A *p*-value < 0.05 was considered as statistically significant.

### 3.2. Serum Triglycerides

**Figure 4:**
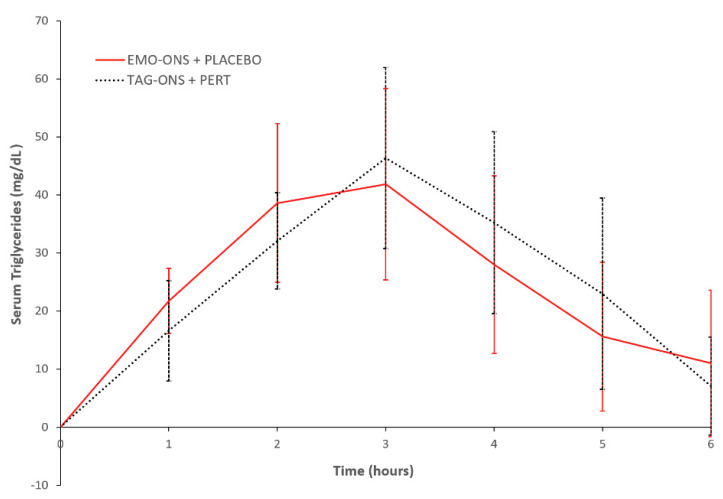
Mean change in serum triglyceride levels. EMO-ONS: enzyme-modified oil oral nutritional supplement. PERT: pancreatic enzyme replacement therapy. TAG-ONS: triacylglycerol-based oral nutritional supplement.

There were no significant differences in the change in serum triglyceride levels between the EMO-ONS+Pert placebo and the TAG-ON+PERT groups (Figure 4). Mean change in peak values from initial serum triglyceride levels at Cmax were not significant between the two groups (41.9 ± 46.7 mg/dL for the EMO-ONS group vs. 46.4 ± 44.1 mg/dL for the TAG-ONS group, two-tailed *p* value = 0.85). The time to achieve peak mean change in serum triglycerides and triglyceride concentrations compared to initial levels was 3 h for both the EMO-ONS and TAG-ONS groups, with mean serum triglyceride levels of 126.5, SEM = ± 39.3 mg/dL vs. 129.0, SEM = ± 25.8 mg/dL respectively; *p* = 0.96 using Wilcoxon Signed Rank Test. There was no difference in median serum triglyceride AUC between the EMO-ONS and TAG-ONS groups with a median AUCEMO-ONS/AUCTAG-ONS ratio for serum triglycerides of 1.36, which is non-significant using Wilcoxon Signed Rank Test (*p* = 0.20).

### 3.3. Serum Glucose

**Figure 5:**
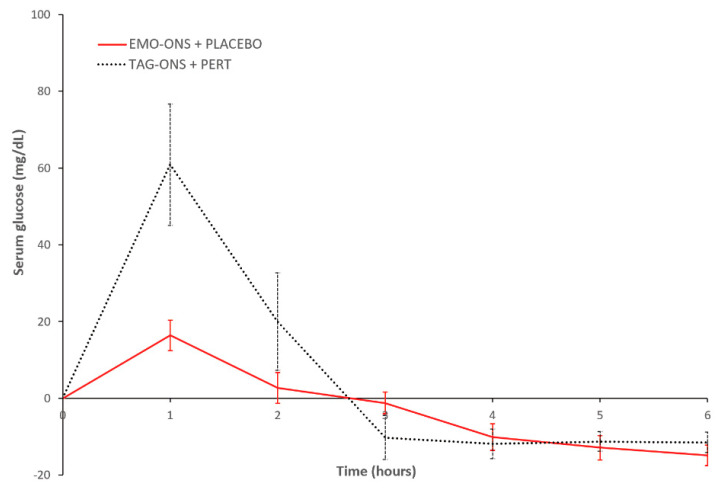
Mean change in serum glucose levels. EMO-ONS: enzyme-modified oil oral nutritional supplement. PERT: pancreatic enzyme replacement therapy. TAG-ONS: triacylglycerol-based oral nutritional supplement.

Figure 5 shows the mean change in serum glucose for the EMO-ONS and TAG-ONS groups over the 6-h study period. Peak concentrations of serum glucose occurred 1 h after initiating dosing in both groups. Serum glucose in the patients who consumed the TAG-ONS initially increased and subsequently decreased to below baseline values over 6 h. There was a much smaller increase in the serum glucose for patients who consumed the EMO-ONS. Mean changes in peak values from initial serum levels at Cmax (1 h) were statistically significant between the two groups (16.4 ± 11.1 mg/dL for the EMO-ONS group vs. 60.9 ± 44.9 mg/dL for the TAG-ONS group, two-tailed *p* value = 0.03). There was a statistically significant difference in median serum glucose AUC between the EMO-ONS and TAG-ONS groups, with a median AUCEMO-ONS/AUCTAG-ONS ratio for serum glucose of 0.28 (two-tailed *p*-value < 0.01).

### 3.4. Other Lipids

Figure S1 shows mean changes in serum lipid levels during the 6-h observation period. There were no significant differences between the TAG-ONS and EMO-ONS groups for any of the lipid parameters studied. For total cholesterol, serum concentration decreased in both groups in the first hour and then gradually increased over the remaining five hours. For the EMO-ONS group, the maximum concentration occurred at 5 h and was slightly elevated over initial values, while the TAG-ONS group had maximum serum cholesterol concentration at 6 h with no change from initial values (2.3 ± 5.7 mg/dL for the EMO-ONS group vs. 0.0 ± 8.6 mg/dL for the TAG-ONS group, two-tailed *p* value = 0.55). No statistically significant differences were found in median serum cholesterol AUC between the EMO-ONS and TAG-ONS.

Serum HDL and LDL concentrations for both groups dropped below initial values and stayed below those initial values over 6 h, except for the maximum serum LDL concentration for the EMO-ONS group, which was slightly elevated above the initial value at 6 h. Peak concentrations of serum HDL and LDL for the EMO-ONS vs. TAG-ONS groups at 6 h are as follows: −0.6 ± 1.6 mg/dL for the EMO-ONS group vs. −1.5 ± 3.9 mg/dL for the TAG-ONS group, two-tailed *p* value = 0.57 and 0.6 ± 3.4 mg/dL for the EMO-ONS group vs. −0.1 ± 3.8 mg/dL for the TAG-ONS group, two-tailed *p* value = 0.68 for HDL and LDL, respectively. As with total serum cholesterol, there were no statistically significant differences in the median serum HDL or LDL AUC between the EMO-ONS and TAG-ONS groups.

As expected, there was an initial increase in serum VLDL levels for both groups, with peak concentrations of serum VLDL occurring at 3 h in both the EMO-ONS and TAG-ONS groups. Maximum concentrations of serum VLDL at 3 h were 8.3 ± 9.1 mg/dL for the EMO-ONS group vs. 9.3 ± 8.7 mg/dL for the TAG-ONS group, two-tailed *p* value = 0.83. Serum VLDL concentrations returned to baseline at the end of 6 h. There were no statistically significant differences in median serum VLDL AUC between the EMO-ONS and TAG-ONS groups.

### 3.5. ONS Tolerance

There were no moderate or severe adverse events reported by subjects during Phase 1 of the study. Two subjects in the EMO-ONS groups reported GI symptoms during the 6 h assessment period (Phase 1). One participant reported severe nausea related to EMO-ONS during Phase 1 of the trial. This subject also reported nausea during the pre-screening session prior to starting the study. The same subject also experienced mild steatorrhea that was reported as being possibly related to EMO-ONS. The subject reported that they had a fatty meal prior to the overnight fast the day before the study. Of note is that this subject did not experience any nausea or steatorrhea when randomized to receive EMO-ONS without the use of pancreatic enzymes for the at-home phase of the study. A total of 3 subjects in the EMO-ONS and 5 subjects in the TAG-ONS group produced stools during the 6 h assessment period with 2 subjects in the EMO-ONS group reporting these were possibly (1 stool) or probably (2 stools), respectively, related to the administration of the ONS. All 5 of the subjects in the TAG-ONS group reported that the stools (range 1 to 3) were not related to ONS administration.

### 3.6. Patient Assessment

Patients preferred the taste, flavor, and aroma of the TAG-ONS compared to the EMO-ONS and rated the volume more filling for TAG-ONS (Supplementary Table S1). While there was a slightly higher rating of the appearance of the TAG-ONS, both products received similar ratings for texture.

### 3.7. Home Trial

Table 2 provides an overview of patient reported outcomes during the 7-day home trial which represented Phase 2 of the study. One subject in the TAG-ONS+PERT group did not return their surveys or diary and they were therefore excluded from the analysis. 

Similar to Phase 1 of the trial, there were no moderate or severe adverse events reported by subjects during Phase 2 of the study. Three subjects in the EMO-ONS group and one subject in the TAG-ONS+PERT group reported events which were possibly related to ONS ingestion. A total of 1patient in the EMO-ONS group described mild pain or bloating over 3 days during Phase 2 of the study. This was followed by 3 days with no reported events for the remaining 4 days of the take-home study with stool consistency and frequency reported as normal. A total of 2 subjects in the TAG-ONS+PERT reported softer stools during the take-home study with 1 reporting having softer and more frequent stools on days 4, 5, and 6 of the study, returning to normal stool frequency and consistency on day 7. The other subject reported they did not take the required PERT capsule with the TAG-ONS on day 3 of the take-home study with resultant steatorrhea and mild abdominal pain which was not attributed to the ONS. No other patients reported a disturbance with stool frequency or consistency over the 7 days for either ONS.

## 4. Discussion

The present proof of concept study demonstrated that EMO-ONS was an effective, well tolerated, and safe nutritional PERT-free alternative to a standard ONS plus PERT in patients with CFI with EPI. EMO-ONS consistently produced similar triglyceride levels when compared to the standard nutritional supplement with PERT with a similar time to peak mean change in triglyceride levels and AUC. Slight elevations in serum triglyceride values compared to baseline were observed 6 h following consumption of the ONS in 7 of the subjects during Phase 1 of the study. This included 3 subjects in the EMO-ONS with PERT placebo group and 4 in the TAG-ONS+PERT group. This observation is consistent with other 6-h lipid tolerance tests that have shown that serum triglyceride levels in most CF patients do not return to baseline over this time period [14], as well as with poor control of triglyceride metabolism and high levels of triglycerides seen in CF patients [15].

As noted above, there was a much greater increase in serum glucose observed for patients who consumed the TAG-ONS compared to when they ingested the EMO-ONS. This difference was likely a result of the much higher amount of sugar in the TAG-ONS product (as glucose syrup and carbohydrates) compared to the EMO-ONS. The mean 460.5 mL volume of the TAG-ONS consumed by patients for the study included an average of 46 g of sugar and 41 g of carbohydrates (which are converted to glucose). This resulted in a mean total glucose consumed of about 87 g for patients in the TAG-ONS group. The serum glucose changes observed for this group resembled a standard glucose tolerance test since the amount of glucose consumed in each TAG-ONS is similar to the amounts in a standard oral glucose tolerance test (100 g). The mean 348.5 mL volume of the EMO-ONS consumed by patients for the study had just 28 g of sugar and approximately 1 g of other carbohydrates; and since this sugar is from date syrup, which is ~28% glucose and ~32% fructose, only approximately 13 g would be detectable in the serum as glucose.

There were no serious adverse events reported for either the inpatient (Phase 1) or outpatient (Phase 2) portions of the study. When reported, GI adverse events were primarily mild and self-limiting with no medical interventions required. One subject in the TAG-ONS+PERT group experienced steatorrhea and mild abdominal pain likely as a result of the admitted failure to take PERT capsules in conjunction with the TAG-ONS.

Adherence with PERT remains a significant issue for patients with CF diagnosed with EPI [11,12]. A recent study reported that adherence to PERT is independently associated with a reduction in hospitalization and length of stay for both children and adults with CF, and it suggested that strategies which improve PERT adherence would improve outcomes in this patient population [13]. The above highlights the potential benefits of a PERT-free alternative like EMO-ONS for patients with CF diagnosed with EPI. The availability of a PERT-free nutritional supplement that eliminates the need for patients to take multiple PERT capsules pre- and post-prandially could result in a reduction in GI side effects associated with the failure to take these capsules, reduced meal skipping, and resultant improvement in nutritional status.

The EMO-ONS is formulated to supply calories, essential fatty acids (omega-3 and omega-6), essential amino acids, and vitamins as a ready-to-drink option patients can consume as preferred, without taking enzyme capsules. While there was a patient preference for TAG-ONS compared to EMO-ONS in regard to both palatability and taste, the formulation of EMO-ONS used for the study had not been optimized for taste or appearance prior to this study with the product. The distributor of the product intends to optimize the taste of the product prior to commercialization while maintaining a similar formulation in terms of lipid profile, carbohydrates, sugars, protein, and vitamin content. This issue may be irrelevant in some tube feeding uses of the ONS.

There are several potential limitations associated with the present study. Since the study was exploratory in nature and since effect size and variability in triglyceride/lipid absorption in this population was unknown, a power analysis was not performed to determine an appropriate sample size. While the small number of patients enrolled in the study limits the ability to generalize its findings, other studies assessing postprandial lipid metabolism, or the use of a crossover study design have also enrolled a similarly small number of patients [14,16]. Additionally, since the study only included patients who were 12 years or older, no conclusions can be made related to the potential safety or beneficial results associated with the use of EMO-ONS in pediatric patients 11 years of age or younger, where PERT adherence and nutritional status are also important.

## 5. Conclusions

The data from this study suggests that an EMO-based ONS may offer a PERT-free alternative to traditional ONS products for patients with CF diagnosed with EPI. Changes in serum triglyceride levels following consumption of EMO-ONS (without PERT) were not significantly different from those observed with TAG-ONS with PERT. The EMO-based ONS product was well tolerated and regarded as palatable by patients participating in this study who represent the targeted patient population for this product. Future well designed clinical studies are needed to document the potential clinical value associated with the use of EMO-ONS

## Figures and Tables

**Table 1:** Patient baseline characteristics and treatment provided for in clinic study.

	Overall (*n* = 10)	Arm 1 (*n* = 5)	Arm 2 (*n* = 5)
Age, years	20.6 ± 28.2	19.3 ± 8.0	22.0 ± 14.9
Weight, kg	54.4 ± 14.3	56.0 ± 12.8	52.8 ± 16.8
Male sex (%)	7 (70%)	4 (80%)	3 (60%)
Lipid delivered, grams	27.2 ± 7.4	28.0 ± 6.4	26.4 ± 8.4
EMO-ONS volume, mL	348.5 ± 94.2	358.7 ± 82.0	338.2 ± 107.4

Data are mean ± Standard Deviation (SD). EMO-ONS: enzyme-modified oil oral nutritional supplement.

**Table 2:** Participant reported outcomes during 7-day at-home study (Phase 2 of trial).

Group	Subject Number	Mild Pain (Events)	Mild Bloating (Events)	Steatorrhea (Events)	Total Days with Events	Possibly Related to ONS	Average Stools per Day	Consistency	Stool Consistency Related to ONS?
TAG-ONS + PERT	100	1-PO	0	0	1	1	2	N	NR
	102	2-NR	0	0	2	0	3	N	PO
	107	1-NR	0	1-NR	1	0	2.4	N	NR
	201	0	0	0	0	0	2	S	NR
EMO-ONS + PERT PLACEBO	101	0	0	0	0	0	2	N	NR
	103	0	0	0	0	0	1.3	N	NR
	104	0	1-PO	0	1	1	2.8	S	PO
	200	3-PO	1-PO	0	3	4	2	N	NR
	202	2-NR	2-NR	0	3	0	2	N	NR

NR: not related. PO: possibly related. Consistency of stools: N = normal/formed, S = soft; EMO-ONS—enzyme modified oil oral nutritional supplement; ONS: oral nutritional supplement. PERT: pancreatic enzyme replacement therapy. TAG-ONS: triacylglycerol oral nutritional supplement.

## Data Availability

Some or all datasets generated and/or analyzed during the current study are not publicly available but they are available from the corresponding author on reasonable request.

## Supplementary Materials

The following are available online at www.mdpi.com/article/10.3390/nu14030680/s1, Table S1: Results from patient palatability survey, Figure S1: Mean change in serum lipid levels for patients in the TAG-ONS + PERT vs. EMO-ONS + placebo over the 6-h study period.
