# Supplementary material for: Intestinal Absorption of Lipids Using a Pancreatic Enzyme-Free Nutritional Supplement in Patients with Cystic Fibrosis: A Randomized, Double-Blind, Crossover Pilot Trial
Source: Nutrients. 2022 Feb 5;14(3):680. doi: 10.3390/nu14030680 (PMC8838800; doi:10.3390/nu14030680)
Supplement: Supplementary file 1 [file nutrients-14-00680-s001.zip › nutrients-1536289-supplementary.pdf]

Table S1. Results from patient palatability survey

	**TAG-ONS** (n=8)	**EMO-ONS** (n=10)
Did you like the taste of the nutritional supplement drink?	3.5	1.1
Did you like the flavor of the nutritional supplement drink?	3.8	1.3
Did you like the aroma (smell) of the nutritional supplement drink?	2.6	1.0
Did you feel the volume of liquid was sufficient for a mid-meal snack, or too little or too much?	4.0	2.8
Did you like the appearance of the nutritional supplement drink?	4.5	3.4
Did you like the texture of the nutritional supplement drink?	3.5	3.2

Based on a scale from 0 to 5, with '0' being least acceptable, to '5' being most acceptable.

EMO-ONS, enzyme modified oil oral nutritional supplement; TAG-ONS, triacylglycerol oral nutritional supplement

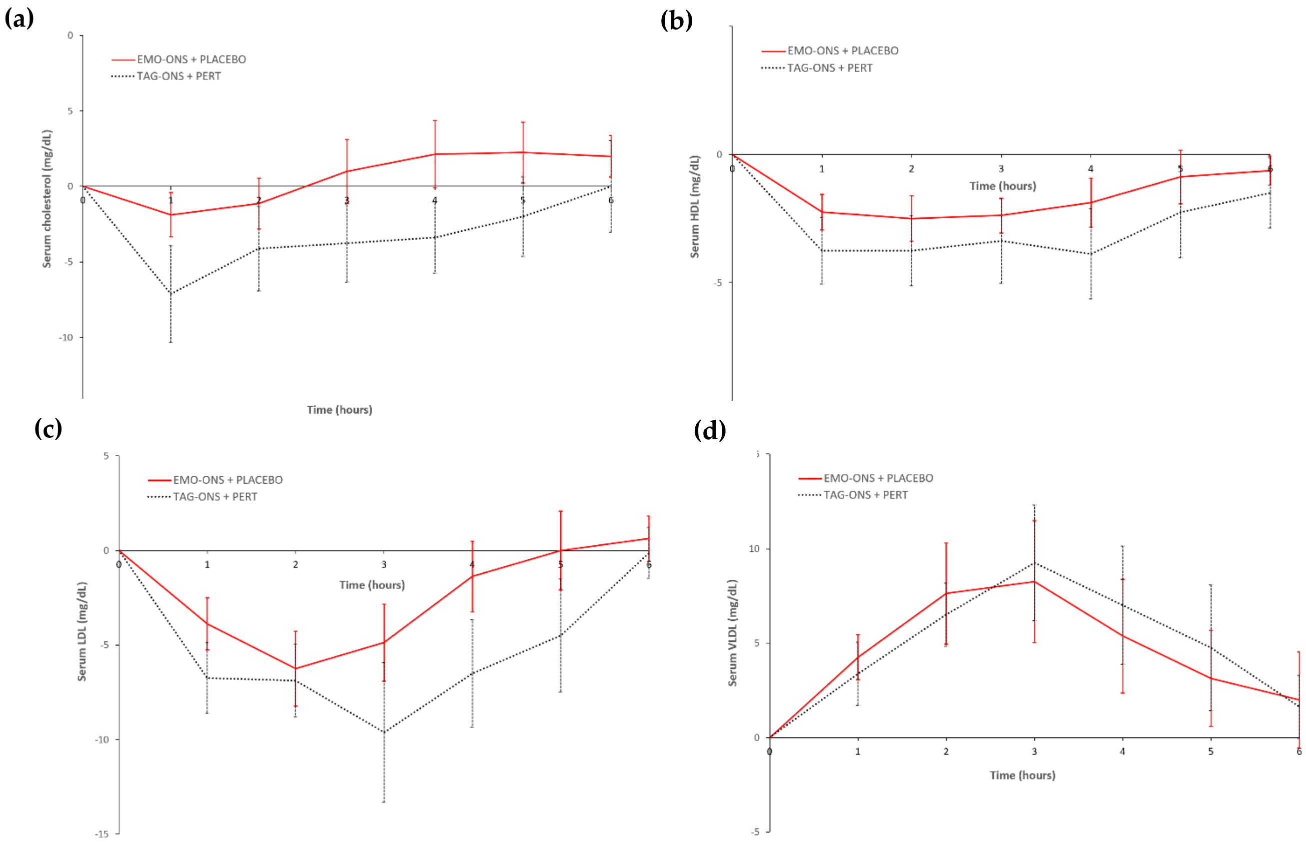

Figure S1. Mean change in serum lipid levels for patients in the TAG-ONS + PERT vs. EMO-ONS + placebo over the 6 hours study period. (a) mean changes in serum cholesterol, (b) mean changes in serum HDL, (c) mean changes in serum LDL, and (d) mean changes in serum VLDL. EMO-ONS: enzyme-modified oil oral nutritional supplement. PERT: pancreatic enzyme replacement therapy. TAG-ONS: triacylglycerol-based oral nutritional supplement.
